# Understanding Ovarian Hypo-Response to Exogenous Gonadotropin in Ovarian Stimulation and Its New Proposed Marker—The Follicle-To-Oocyte (FOI) Index

**DOI:** 10.3389/fendo.2018.00589

**Published:** 2018-10-17

**Authors:** Carlo Alviggi, Alessandro Conforti, Sandro C. Esteves, Roberta Vallone, Roberta Venturella, Sonia Staiano, Emanuele Castaldo, Claus Yding Andersen, Giuseppe De Placido

**Affiliations:** ^1^Department of Neuroscience, Reproductive Science and Odontostomatology, University of Naples Federico II, Naples, Italy; ^2^Istituto per l'Endocrinologia e l'Oncologia Sperimentale Consiglio Nazionale Delle Ricerche, Naples, Italy; ^3^ANDROFERT, Andrology and Human Reproduction Clinic, Campinas, Brazil; ^4^Unit of Obstetrics and Gynaecology, Department of Experimental and Clinical Medicine, Magna Graecia University of Catanzaro, Catanzaro, Italy; ^5^Laboratory of Reproductive Biology, Faculty of Health and Medical Sciences, University Hospital of Copenhagen, Copenhagen, Denmark

**Keywords:** hypo-response, ovarian stimulation, ovulation induction, assisted reproductive technology, *in vitro* fertilization, follicle to oocyte index, follicle output rate, POSEIDON criteria

## Abstract

Hypo-responsiveness to controlled ovarian stimulation is an undervalued topic in reproductive medicine. This phenomenon manifests as a low follicles output rate (FORT) with a discrepancy between the relatively low number of pre-ovulatory follicles which develop following ovarian stimulation as compared to the number of antral follicles available at the start of stimulation. The pathophysiology mechanisms explaining the ovarian resistance to gonadotropin stimulation are not fully understood, but the fact that both hypo-responders and normal responders share similar phenotypic characteristics suggests a genotype-based mechanism. Indeed, existing evidence supports the association between specific gonadotropin and their receptor polymorphisms and ovarian hypo-response. Apart from genotypic trait, environmental contaminants and oxidative stress might also be involved in the hypo-response pathogenesis. The ratio between the number of oocytes collected at the ovum pick up and the number of antral follicles at the beginning of OS [Follicle to oocyte index (FOI)] is proposed as a novel parameter to assess the hypo-response. Compared with traditional ovarian reserve markers, FOI might reflect most optimally the dynamic nature of follicular growth in response to exogenous gonadotropin. In this review, we contextualize the role of FOI as a parameter to identify this condition, discuss the underlying mechanisms potentially implicated in the pathogenesis of hypo-response, and appraise possible the treatment strategies to overcome hyper-responsiveness to gonadotropin stimulation.

## Introduction

Ovarian stimulation (OS) is an essential step in assisted reproductive technology (ART). The conventional OS approaches lead to sufficient follicular growth and proper estrogen levels in the majority of women. In this regard, the number of mature oocytes retrieved is the parameter most often used to assess ovarian response to exogenous gonadotropin, as oocyte number is closely related to the likelihood of achieving a live birth in ART (1). Based on oocyte number, women are usually classified as poor, suboptimal, normal or hyper-responders (2, 3). Outside these categories, a subgroup of women with impaired response to gonadotropin, termed “hypo-responders,” also exists. An unexpected ovarian resistance to OS with use of standard age- and BMI-matched doses of exogenous FSH characterizes these patients (4–6). The clinical manifestation of ovarian resistance includes either an “initial slow response” to FSH stimulation concerning estradiol levels rise and follicle growth (7, 8) or can be retrospectively diagnosed in women who require higher-than-expected doses of gonadotropins considering their age, BMI, and ovarian reserve (9). In contrast to “suboptimal response,” which is based essentially on the number of oocytes retrieved (between 4 and 9) (10), the hypo-response profile refers to those patients who show a resistance to gonadotropin stimulation and in which the number of oocytes retrieved at the end of stimulation is not consistent with the number of antral follicle count (AFC) available at the beginning of OS. In this review, we (1) illustrate how to identify patients with ovarian resistance to exogenous gonadotropins who undergo ART by use of a new marker named follicle-to-oocyte index (FOI), (2) discuss the underlying pathogenetic mechanisms associated with hypo-response, and lastly, (3) critically appraise possible treatment strategies to overcome this condition.

## Assessment of hypo-response

The prediction of ovarian response is crucial for an optimal and individualized management in the context of OS. It also allows clinicians to better counsel women about the risk of adverse events following OS, such as protracted cycles, cycle cancellation due to poor ovarian response, or ovarian hyperstimulation syndrome (OHSS). Generally, the ovarian response to gonadotropin stimulation can be explained by the interplay between demographic and anthropometric characteristics, and the individual's ovarian reserve. In this regard, biological (AFC) and biochemical [Anti-Müllerian hormone (AMH)] markers have been introduced to predict both the poor and hyper response with fairly good accuracy (3).

**Figure 1:**
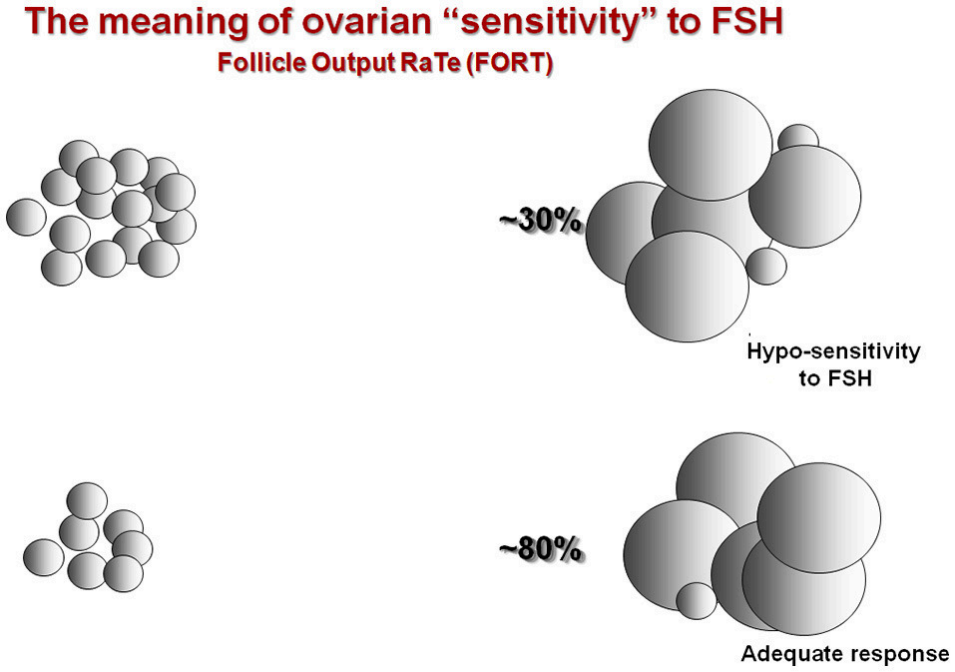
Ovarian sensitivity using FORT [adapted from (12)].

In our opinion, these biomarkers represent a “static” snapshot of the individual ovarian reserve which do not properly reflect the “dynamic” nature of follicular growth in response to exogenous OS. An interesting model to assess hypo-responsiveness during OS is the follicle output rate (FORT) introduced by Genro et al. in 2011 (11). This index is calculated as the ratio between the number of pre-ovulatory follicles obtained in response to OS with FSH administration and the pre-existing pool of small antral follicles (11, 12). A low FORT (e.g., 30%) indicates hypo-response, due to the discrepancy between the relatively low number of pre-ovulatory follicles which develop following OS as compared to the number of antral follicles available at the start of stimulation (Figure 1). Notably, low FORT indices are not associated with reduced ovarian markers, thereby suggesting that patients undergoing OS can present with a low FORT despite the presence of adequate ovarian markers (11).

Another parameter that might be used to assess hypo-responsiveness is the ovarian sensitivity index (OSI) (13). OSI is calculated by dividing the total administered FSH dose and the number of retrieved oocytes. A high OSI index reflects ovarian resistance to OS thereby suggesting a hypo-response profile.

Although both methods seem to be useful in the evaluation of hypo-response, some drawbacks should be considered. The FORT does not assess the actual number of oocytes retrieved, which is the parameter more strictly associated with live birth rates (1). On the other hand, OSI does not take into account the type of gonadotropin adopted (recombinant or urinary) nor does it consider the gonadotropin regimen utilized. In fact, recent evidence indicates that the use of luteinizing hormone (LH) or LH-like activity during OS improves follicle development in specific subgroups of women, including hypo-responders (14, 15). Along the same lines, OSI indices might be misleading if inappropriate low starting doses of exogenous gonadotrophins are given. Lastly, it has been suggested that OSI results are associated with AMH levels in women undergoing IVF (13), thus making it a less robust index to assess the dynamical aspect of the follicular response to OS.

## Follicle-to-oocyte index (FOI)

**Figure 2:**
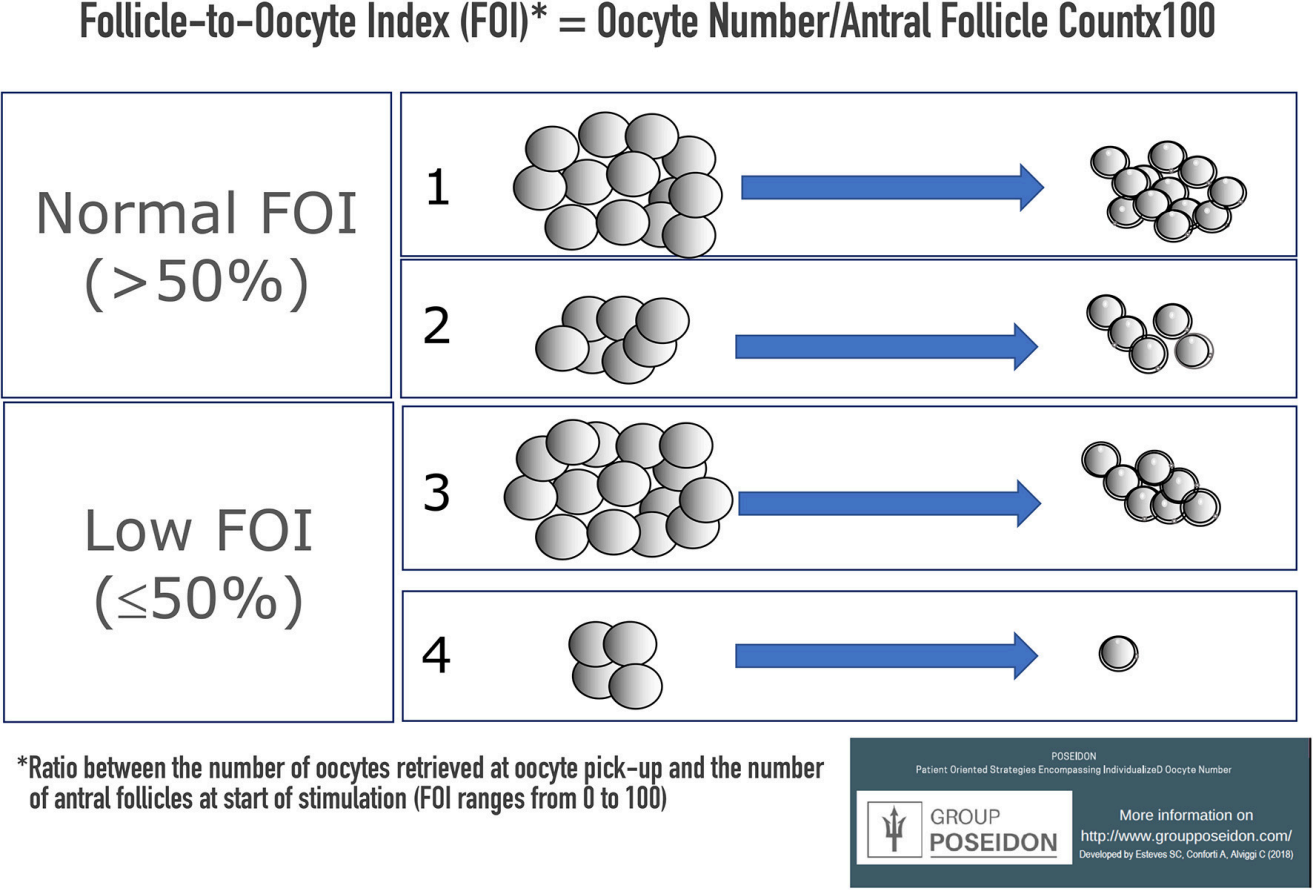
Ovarian sensitivity using the Follicle-to-Oocyte Index (FOI). Case number 1 depicts a patient with normal FOI, in whom the number of oocytes retrieved was consistent with the AFC at the start of stimulation. Case number 2 illustrates a patient with suboptimal number of oocytes retrieved (between 4 and 9), but with a normal Follicle-to-Oocyte index (FOI >50%). Case number 3 shows a patient with both hypo-response and suboptimal oocyte number. This patient had only 7 oocytes collected despite an AFC of 15 at the beginning of stimulation (FOI ≤ 50%). Case number 4 depicts a patient with both hypo-response and poor response.

**Figure 3:**
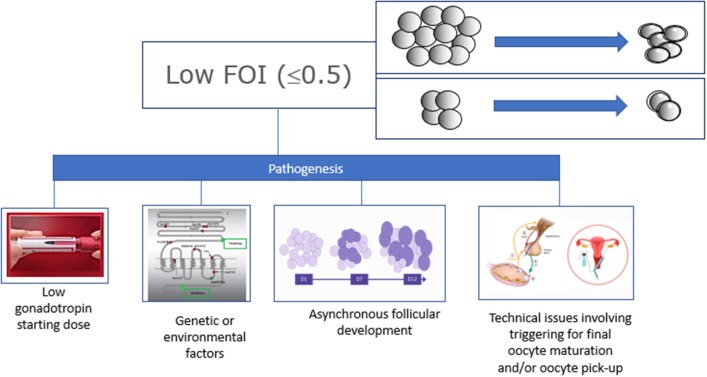
Possible causes of low Follicle-to-Oocyte Indeces.

We propose an alternative approach to address the ovarian resistance to gonadotropin stimulation (or hypo-responsiveness) based on the concept of FORT, namely, the ratio between the total number of oocytes collected at the end of OS, and the number of antral follicles available at the start of stimulation (Follicle-to-Oocyte Index [FOI]) (Figures 2, 3). Figure 2 illustrates the difference between hypo-response to OS and suboptimal response. In detail, both cases 2 and 3 show a suboptimal response with the number of oocytes retrieved between 4 and 9. However, only case 3 illustrates a hypo-response profile in which just 7 oocytes were collected despite an AFC of 15 at the beginning of stimulation (FOI < 50%). On the other hand, in case 2, despite the low oocyte number, this hypothetical woman had 5 oocytes retrieved from an AFC of 7, thus illustrating a normal follicle-to-oocyte index (FOI >50%). Lastly, case number 4 depicts a patient with both hypo-response and poor response. Based on the examples above it is therefore clear that hypo-responsiveness and suboptimal/poor response are not synonymous.

FOI may be used alone or combined with FORT to most optimally reflect the ovarian resistance to OS. The results of FOI can also help to understand whether it is possible to exploit the ovarian reserve further using pharmacologic interventions. Lastly, FOI could be useful to predict the likelihood of success in ART, both concerning the chances of achieving at least one euploid blastocyst for transfer in each patient–the so-called POSEIDON marker of successful outcome-, (4, 6) as well as pregnancy success. Thus, low FOI values imply that only a fraction of available antral follicles was exploited during OS, suggesting that there might be therapeutic opportunities to change the fate of these women in a subsequent OS. Naturally, technical aspects related to oocyte retrieval and triggering for final oocyte maturation, both of which can influence FOI results, should be taken into account in patients with low FOI. The FOI is under evaluation by an ongoing multicenter Italian study (Impact of **G**onadotropin **GEN**etics Profile and **O**v**A**rian **R**eserve on **C**ontrolled **O**varian Stimulation, the **GENACOS** study). In future, we envision refining FOI by including the amount of gonadotropin used during OS. Additionally, a prediction model can be developed to estimate the likely number of oocytes to be retrieved at the end of OS by computing the results of AFC, polymorphisms of gonadotrophins and their receptors, and FSH starting dose.

## Pathogenesis of hypo-response

The pathophysiology mechanisms explaining hypo-responsiveness to OS are not fully understood. However, the link between ovarian response and individual genotype has been postulated by several authors (16–22). Furthermore, the fact that both hypo-responders and normal responders share similar phenotypic characteristics suggests a genotype-based mechanism (23–26, 27). In other words, hypo-responders might have a particular genotype profile which influences their response to OS (28). Indeed, several studies support this concept. In a 2013 sizeable longitudinal study, we found that a common LH beta subunit variant was associated with increased FSH consumption during OS (29). In another study, we found that the prevalence of hypo-response was higher in G allele carriers of a common FSH receptor (FSHR) polymorphism (p.N680SA > G, rs6166) than in wild-type haplotypes (30). Furthermore, *in vitro* studies using human granulosa cells demonstrated that p.N680SA>G G homozygous showed higher resistance to FSH stimulation than p.N680SA>G A homozygous carriers at the FSH receptor level (31, 32). Along the same lines, it has been shown that N680SA>G G homozygous display increased basal endogenous FSH levels (33) compared to p.N680SA>G A homozygous, thus corroborating the hypothesis of an impaired FSHR function in carries of G allele. Added to this, *in vivo* studies have shown that carriers of another FSHR polymorphism, namely, A allele, have ovarian resistance to OS as expressed by a higher consumption of exogenous gonadotropin than G allele carriers (34, 35). It is out of the scope of our paper to provide readers a comprehensive review of the impact of gonadotropin receptors polymorphisms in OS, but a recently published systematic review and meta-analysis by our group confirmed that polymorphism of FSHR could impair ovarian response to exogenous gonadotropin (28).

**Figure 4:**
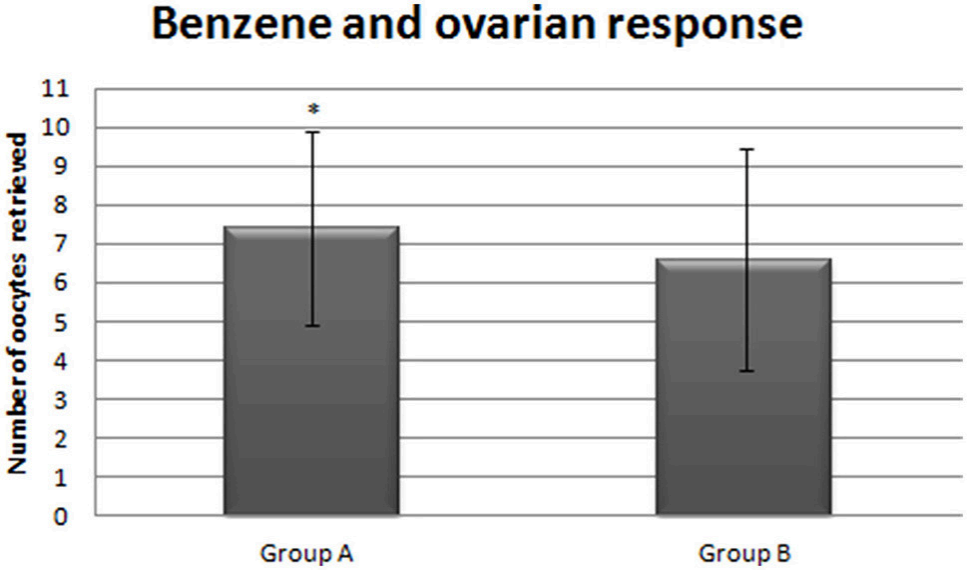
Number of oocytes collected form patients with low (Group A) vs. high intrafollicular benzene levels [adapted from (36)]. *stand for statistically significant.

Apart from the genotypic trait, there is also evidence that environmental contaminants might influence ovarian response to gonadotropin stimulation. In a 2014 retrospective study, we showed that elevated intra-follicular levels of benzene were associated with a reduced number of oocytes retrieved and embryos available for transfer in women who underwent IVF (36) (Figure 4). The mechanism underlying this phenomenon is not clear, but the authors hypothesized that the toxic effect of benzene leads to a transduction deficiency of the FSHR. In fact, this hypothesis is supported by the fact that basal FSH levels were significantly higher in women with higher intra-follicular benzene levels than in women with low intra-follicular benzene levels (36). Other pollutants were also associated with an impaired ovarian response in IVF (37). In a 2017 retrospective study, a significant inverse association was found between the levels of polychlorinated biphenyl congeners (PBC) in follicular fluid of women undergoing ART and the ovarian response to gonadotropins measured by both the number of oocyte retrieved and estradiol levels (38). Notably, the number of antral follicles also seem to be affected by the levels of PCB congeners in follicular fluid (38).

Lastly, accumulating evidence indicates that oxidative stress might also affect both folliculogenesis and spermatogenesis (39, 40). In detail, it was hypothesized that oxidative stress and excessive free radicals such as reactive oxygen species (ROS) might influence the quality of oocytes, spermatozoa, and embryos, as well as their environments (41), thus negatively affecting the outcome of IVF (39, 42). In a 2016 pilot study involving women with polycystic ovarian syndrome (PCOS) (43), in whom oxidative stress seemed to be a relevant pathogenetic factor, we demonstrated that myo-inositol plus active antioxidants (glutathione, selenium, vitamins C and E, and zinc), given twice a day for 5 months preceding OS, had a favorable effect on the outcome of IVF by increasing the number of mature oocytes (44). Recently, in 2018, Xu et al. reported the results of a randomized controlled trial with the use of coenzyme Q10 as a pretreatment to OS in patients with low prognosis in ART (45). The authors utilized the POSEIDON criteria to enroll young patients (<35 years-old; POSEIDON group 3) with poor ovarian reserve parameters (4, 6). In their study, the use of coenzyme Q10 (200 mg thrice daily for 60 days preceding the IVF cycle) was associated with an increased number of retrieved oocytes, fertilization rate, and high-quality embryos than in non-treated women. Lastly, a 2017 Cochrane meta-analysis supports the above observations by showing that antioxidant intake might provide a benefit for subfertile women who undergo ART (42). Whether oxidative stress has a role in the pathogenesis of hypo-response deserves further investigation.

## Clinical management of patients with hypo-responsiveness to ovarian stimulation

In clinical practice, ovarian resistance to gonadotropin stimulation is still a largely undervalued issue. Overall, clinicians do not ask themselves whether or not the number of oocytes retrieved after OS was consistent with the patient's potential based on the results of AFC at the start of stimulation. In our opinion, the number of oocytes retrieved should be interpreted in the light of an individual ovarian reserve. For example, in a woman with an AFC of 12, recruitment of 7 oocytes, which is far above the adopted POR threshold (4, 46), might still denote an inappropriate ovarian response to stimulation. Nevertheless, very few trials have investigated the role of interventions in women with ovarian resistance (i.e., hypo-responsiveness) to OS and until recently no practical guidelines were available.

An increase in the daily dose of exogenous FSH represents the intuitive approach to overcome ovarian resistance to exogenous FSH, and it was indeed adopted by several investigators. This strategy might be applied to rescue an ongoing OS cycle in women with an initial slow (“steady”) response to gonadotropin stimulation (7, 8, 47). Increased FSH dosages has been mostly utilized in women treated with GnRH-a long protocols, where follicle “stagnation” during the first days of OS is more frequently detected. The increase in the FSH starting dose might be also an option in women who show hypo-sensitivity to gonadotropin stimulation in a previous cycle. In the latter, use of higher dosages of recombinant FSH might mitigate the negative effect of FSHR polymorphisms on ovarian response. In one study, Behre et al. demonstrated that increasing the daily FSH dose might counteract the negative effect of FSHR polymorphisms in normogonadotropic women with p.N680SA > G, rs6166 haplotype. In their study, the recombinant FSH dose of 225 IU/day was able to prevent low estradiol levels achieved at the end of OS in p.N680SA>G G homozygous stimulated with 150 IU/day (16). These results were corroborated by a 2012 study conducted by Genro et al. The authors reported that the FORT was not significantly influenced by the presence of FSHR p.N680SA > G, rs6166 polymorphism when a high FSH dose (300 IU per day) was given during OS (48).

Based on the aforementioned observations, one could argue that a starting FSH dose between 225–300 IU should be considered for all good prognosis patients undergoing ART, independently of genotype characterization. Although this approach might counteract the vast majority of the polymorphisms of gonadotrophins and their receptors, it is clearly not cost-effective. The study by Behre et al. (16) mentioned above indicates that a remarkable proportion of untested women would achieve an optimal FORT with a lower FSH starting dose. In addition, the MERIT study demonstrated that the indiscriminate use of a 225 IU/day FSH starting dose led to progesterone rise in a relevant percentage of women with good ovarian reserve (49). In this study, progesterone elevation was not observed if a starting dose of 150 IU/day had been adopted (50). Hence, FSHR genotype testing before OS might be clinically useful and cost-effective to identify those women who benefit from an increment in the FSH starting dose from those who do not (28).

The use of recombinant LH (r-hLH) supplementation has also been investigated as a means to overcome hypo-responsiveness to gonadotropin stimulation (51–53). Recently, a systematic review compiled the evidence concerning the use of r-hLH supplementation in hypo-responder women undergoing IVF (15). From the analysis of RCTs, the authors concluded that addition of rLH might be more advantageous than increasing rFSH dosage. Notwithstanding the promising results with the use of r-LH in hypo-responders, the existing literature is limited by the availability of only few reports, thus indicating the need of further research. Likewise, the use antioxidant supplementation as a means of alleviating the plausible negative effects of ROS on the follicular environment and ovarian resistance to gonadotropin stimulation is open for research.

## Conclusion

Several non-mutually exclusive factors seem to influence ovarian resistance to gonadotropin stimulation. The driving theory explaining its pathophysiology relies on the genotypic profile of gonadotropins and their receptors. Genetic phenotyping of relevant polymorphisms seems to be the optimal method to identify these patients. Until genotyping testing becomes widely available, other indices such as the FORT and FOI can be used as surrogate measures to identify women with ovarian resistance (hypo-responsiveness) to gonadotropin stimulation. Particularly, FOI, assessing the actual number of oocytes retrieved could represent a better tool to determine whether the ovarian reserve was adequately exploited during stimulation. Guidance on how to most optimally manage patients with hypo-response to OS is lacking, but limited evidence indicates that the use of higher FSH daily doses alone or combined with recombinant LH supplementation are the most effective ways to counteract the negative effects of hypo-responsiveness to exogenous gonadotropin administration. Further research is warranted to fully unravel the underlying mechanisms leading to ovarian resistance to gonadotropin stimulation and to determine the most prevalent polymorphisms associated with this condition. Additionally, the impact of different pharmacological regimens as a means of overcoming ovarian resistance to gonadotropin stimulation needs to be investigated in more detail.

## Author contributions

CA, AC, SE, CYA, and GD idealized the paper and wrote the first draft. RVa, RVe, SS, and EC participated in literature research and paper writing. All author listed have made intellectual contribution to the work and approved the final version.

### Conflict of interest statement

CA, AC, SE, CYA, and GD are Poseidon members. The remaining authors declare that the research was conducted in the absence of any commercial or financial relationships that could be construed as a potential conflict of interest. The reviewer GB declared a past co-authorship with several of the authors SE, CA, and AC to the handling editor.
